# Immune checkpoint imbalance in ANCA-associated vasculitis: insights into disease activity and precision immunotherapy

**DOI:** 10.3389/fmed.2026.1773241

**Published:** 2026-03-02

**Authors:** Lydia García-Serrano, Laura Martinez-Valenzuela, Juliana Draibe

**Affiliations:** 1 Immunology Department, Hospital Universitari de Bellvitge, L’Hospitalet de Llobregat, Spain; 2 Bellvitge Biomedical Research Institute (IDIBELL), L'Hospitalet de Llobregat, Spain; 3 Facultat de Medicina i ciències de la salut, Universitat de Barcelona, Barcelona, Spain; 4 Nephrology Department, Hospital Universitari de Bellvitge, L’Hospitalet de Llobregat, Spain

**Keywords:** ANCA-associated vasculitis, biomarkers, immune checkpoint, immunology, precision immunotherapy

## Abstract

Anti-neutrophil cytoplasmic antibody (ANCA)–associated vasculitis (AAV) is a systemic autoimmune disease characterized by necrotizing small-vessel inflammation, in which dysregulated adaptive immune responses play a central pathogenic role. Beyond the well-established contribution of ANCAs and innate immune activation, increasing evidence highlights profound alterations in T-cell regulation that drive persistent inflammation, autoantibody production, and organ damage. Immune checkpoints (ICs)—a network of co-stimulatory and co-inhibitory pathways that fine-tune lymphocyte activation and maintain peripheral tolerance—have emerged as key regulators in this process. In this review, we summarize current experimental and clinical evidence demonstrating imbalance across multiple immune checkpoint pathways in AAV, including the PD-1/PD-L1/PD-L2 axis, CD28/CTLA-4, ICOS, CD40–CD40L, OX40, LAG-3, TIM-3, BTLA, and CD27. We discuss how impaired inhibitory signaling combined with enhanced co-stimulatory activity promotes sustained T-cell activation, aberrant T–B cell collaboration, and pathogenic ANCA production, contributing to vascular and renal injury. Importantly, both membrane-bound and soluble checkpoint molecules show disease-specific alterations in blood, urine, and renal tissue, correlating with disease activity, renal involvement, treatment response, and relapse risk. These findings position immune checkpoint components as promising biomarkers that may complement conventional clinical and serological markers. Finally, we review the current therapeutic landscape of checkpoint modulation in AAV, including clinical experience with abatacept and emerging evidence supporting PD-1 agonism and other pathway-targeted strategies derived from related autoimmune diseases. Collectively, this work highlights immune checkpoint dysregulation as a central feature of AAV pathophysiology and underscores its potential for advancing precision biomarkers and immune-targeted therapies.

## 1 Introduction

Anti-neutrophil cytoplasmic antibody (ANCA)–associated vasculitis (AAV) is a systemic autoimmune disease characterized by necrotizing small-vessel vasculitis, predominantly affecting the kidneys and the respiratory tract. Patients develop autoantibodies directed against neutrophil and monocyte granule proteins, mainly proteinase 3 (PR3) and myeloperoxidase (MPO) (1). Based on clinical and pathological features, AAV is classified into three main subtypes: granulomatosis with polyangiitis (GPA), microscopic polyangiitis (MPA), and eosinophilic GPA (EGPA). GPA is more often associated with PR3-ANCA, whereas MPO-ANCA is common in MPA and EGPA (2, 3). AAV is considered a rare disease, with an estimated global prevalence of approximately 200 cases per million persons (4, 5).

While ANCAs are well established as key serological markers of AAV, the mechanisms linking them to disease development are not fully understood. Current evidence indicates that genetic, environmental, and infectious factors contribute to a proinflammatory environment that primes neutrophils and monocytes, leading to surface expression of PR3 and MPO and subsequent ANCA binding (6). This interaction triggers cell activation, degranulation, production of reactive oxygen species, and formation of neutrophil extracellular traps (NETs) (7), ultimately driving vascular injury. In addition, activation of the alternative complement pathway, particularly via C5a-mediated neutrophil recruitment and activation, exacerbates tissue damage and sustains disease activity (8).

Beyond innate immune mechanisms, adaptive immune responses, particularly T cells, also play a crucial role in AAV. Patients display a shift from naïve to effector memory CD4^+^ subsets, with persistent activation of Th1 and Th17 populations in both blood and tissue lesions (9). In addition, regulatory T cells (Tregs) show impaired function and may adopt proinflammatory phenotypes under chronic inflammation, further contributing to loss of tolerance. Similarly, CD8^+^ T cells exhibit clonal expansion and transcriptional profiles consistent with sustained activation, which have been associated with relapse and poor renal prognosis (10). Experimental models further support the pathogenic relevance of T cells, as depletion of either CD4^+^ or CD8^+^ subsets reduces glomerulonephritis and improves outcomes (11).

These alterations highlight the importance of regulatory pathways controlling T-cell activity. Immune checkpoints (ICs) constitute a family of co-stimulatory and co-inhibitory molecules that prevent excessive lymphocyte activation, maintain tolerance, and avoid autoimmunity. Evidence from experimental and clinical studies indicates that alterations in these pathways may contribute to AAV pathogenesis. Importantly, despite the availability of current therapies, AAV is still characterized by a high incidence of relapse, and established biomarkers, such as ANCA titers, show limited reliability in predicting disease flares. In this context, ICs have emerged as candidate biomarkers of disease activity and relapse risk, as well as potential therapeutic targets. This review summarizes current knowledge on immune checkpoints in AAV, focusing on their role in pathogenesis, their value as biomarkers, and their potential as therapeutic targets. Details on the literature search strategy are provided in Supplementary Box S1.

## 2 Overview of the different immune checkpoint pathways and their role in AAV pathogenesis

T cell activation requires not only antigen recognition through the T-cell receptor (TCR) but also co-stimulatory and co-inhibitory signals provided by ICs. These molecules are predominantly expressed as surface proteins on T cells, B cells, natural killer (NK) cells, antigen-presenting cells (APC), and certain non-immune cells, highlighting their broad role in immune regulation.

**Figure 1:**
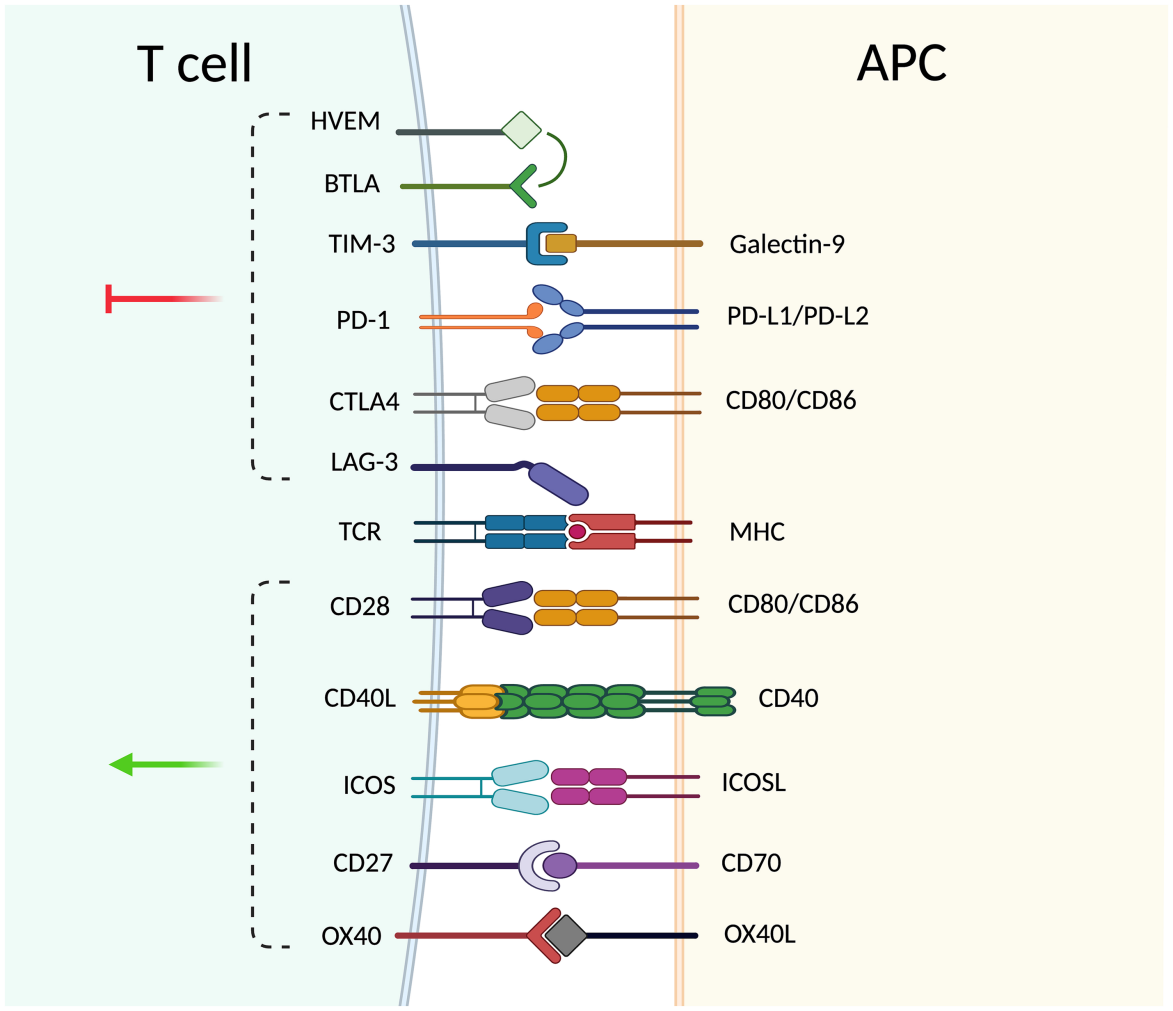
Schematic representation of co-stimulatory and co-inhibitory interactions between a T cell and an antigen-presenting cell (APC, antigen-presenting cell). Major co-stimulatory pathways include CD28–CD80/CD86, ICOS (inducible T-Cell co-stimulator)–ICOSL (ICOS ligand), OX40 (CD134; Ox40 receptor)–OX40L (OX40 ligand), CD27–CD70, and CD40L (CD154)–CD40. Co-inhibitory pathways include PD-1 (programmed cell death protein 1)–PD-L1/PD-L2 (programmed death-ligand 1/2), CTLA-4 (cytotoxic T-lymphocyte–associated protein 4)–CD80/CD86, LAG3 (lymphocyte-activation gene 3)–MHC class II (major histocompatibility complex class II), TIM3 (T-cell immunoglobulin and mucin-domain containing-3)–galectin-9, and the BTLA–HVEM axis (BTLA, B and T lymphocyte attenuator; HVEM, herpesvirus entry mediator). Positive signals (green arrow) promote T-cell activation, whereas negative signals (red arrow) regulate and inhibit T-cell responses. Antigen recognition through the T-cell receptor (TCR, T-cell receptor) and MHC class II on the APC is also shown. Created in https://BioRender.com.

Functionally, ICs can be classified into co-stimulatory and co-inhibitory receptors. Co-stimulatory molecules promote proliferation, differentiation, and survival through interactions. Inhibitory pathways inhibit T-cell activation and contribute to the maintenance of peripheral tolerance (12). The equilibrium between these signals is critical to prevent excessive immune activation and the development of autoimmunity. Figure 1 provides an overview of the major immune checkpoint pathways, and Table 1 summarizes the key observations reported for each checkpoint axis in AAV, including alterations in cellular expression and soluble ICs components. In addition to their membrane-bound forms, ICs also exist as soluble isoforms generated either by alternative splicing or by proteolytic cleavage of extracellular domains. Soluble ICs can be detected in biological fluids and may exhibit a dual role: while they can retain the same co-stimulatory or inhibitory functions as the transmembrane form, they may also modulate immune signaling by interfering with receptor–ligand interactions. However, their physiological roles remain incompletely understood.

**Table 1:** Summary of immune checkpoint and co-stimulatory biomarkers evaluated in ANCA-associated vasculitis (AAV), including biological specimens, disease-activity comparisons, and associations with clinical and pathological outcomes.

Biomarker	Specimen	Status	Comparison group	Clinical/pathological correlation	Ref
PD-1	PD-1 + Tph cells	↑	Active vs. remission	Decreases after immunosuppression.	(22)
sPD-1	Serum	↑	Active vs. HC	Correlates with BVAS and inflammation.	(33)
PD-L1	Monocytes	↓	Active vs. HC	Correlates with higher ANCA titters	(21)
sPD-L1	Serum	↑	Active vs. HC	Decreases with treatment.	(33)
sPD-L2	Serum	↓	Active vs. remission	Higher in remission than in active disease.	(33)
PD-1/PD-L1	Renal tissue	↓	AAV vs. HC	Correlates with active glomerular and interstitial lesions.	(23, 69)
uPD-1/ uPD-L2	Urine	↓	Active vs. HC	Lowest in acute phase; recovers in remission.	(70)
sCD28	Serum	↑	Active vs. HC	Correlates with BVAS and inflammation.	(33)
sCTLA-4	Serum	↓	AAV vs. HC	Declines further during remission (parallel to BVAS).	(33)
ICOS	ICOS+ Tph cells	↑	AAV vs. HC		(42)
sICOS	Serum	↑	AAV vs. HC	Reflects systemic enhancement of ICOS signaling.	(33)
sCD40L	Serum and surface	↑	AAV vs. HC		(48)
sCD40	Serum	=	AAV vs. HC	Correlates with renal dysfunction.	(33)
OX40	Blood/inflamed tissue	↑	AAV vs. HC	Predominantly TNF-α producing cells.	(54, 55)
sLAG-3	Serum	↑	AAV vs. HC	Combination of lower levels of sLAG-3 and higher levels of sCD27 predict RTX therapy failure.	(33, 74)
sTIM-3	Serum	↑	AAV vs. HC	Correlates with renal parameters; high baseline values correlates with sustained remission.	(33, 74)
sBTLA	Serum	↓	Active vs. remission	Correlates with BVAS. High baseline values correlates with sustained remission.	(33, 74)
CD27	CD27 + B cells	↑	Acute vs. remission	Correlates with renal involvement.	(68)
sCD27	Serum	↑	AAV vs. HC	Combination of lower levels of sLAG-3 and higher levels of sCD27 predict RTX therapy failure.	(33, 74)

Although PD-1/PD-L1/PD-L2 represents the best-characterized pathway, other checkpoints are also likely to play a role in AAV. Studies have reported altered expression of additional inhibitory receptors, including CTLA-4, TIM-3, LAG-3, and TIGIT, as well as changes in co-stimulatory pathways such as ICOS and CD27, particularly in their soluble forms. While mechanistic data remain limited, these findings suggest that dysregulation extends beyond the PD-1 axis and that imbalance across multiple checkpoint pathways may collectively contribute to the breakdown of tolerance in AAV. Taken together, IC operate as integrated regulators of peripheral tolerance by constraining autoreactive clones, supporting regulatory networks, and limiting effector activity in peripheral tissues.

### 2.1 PD-1/PD-L1/PD-L2 axis

PD-1 is an inhibitory receptor of the CD28 family that is inducibly expressed on T cells, B cells, and NK cells, and is also expressed in subsets of myeloid cells (13). Its ligands, programmed death-ligand 1 (PD-L1) and programmed death-ligand 2 (PD-L2), show distinct but complementary patterns of expression. PD-L1 is broadly expressed at low levels on APCs and on a broad range of non-hematopoietic tissues such as endothelial and epithelial cells. Its expression is strongly induced by inflammatory cytokines, particularly type I and type II interferons. PD-L2 expression is more restricted, largely confined to dendritic cells and macrophages, and is upregulated by IL-4 and GM-CSF (14).

Upon ligand binding, the cytoplasmic tail of PD-1 recruits the phosphatases SHP-1 and SHP-2 through its immunoreceptor tyrosine-based inhibitory motif (ITIM) and switch motif (ITSM). SHP-2 dephosphorylates key signaling molecules such as CD3ζ and ZAP70, which leads to reduced TCR activation. This also inhibits PI3K signaling, leading to reduced interleukin-2 (IL-2) production and impaired metabolic activity (15). The resulting IL-2 deficiency promotes anergy in both CD4^+^ and CD8^+^ T cells (16). Inhibitory signals mediated by PD-1 and its ligands (PD-L1/PD-L2) contribute to the regulation of central and peripheral tolerance through various mechanisms. In the thymus, PD-1 expression is initiated in CD4 − CD8 − double-negative thymocytes as they undergo TCRβ rearrangement, where it fine-tunes signaling thresholds for positive selection, thereby limiting the expansion of double-positive thymocytes and contributing to central tolerance (17). At the peripheral level, PD-1 signaling depends on the differentiation state of the T cell: in naïve T cells, PD-1 ligation increases the activation threshold. This restricts proliferation and differentiation after antigen stimulation and thereby contributes to the maintenance of peripheral tolerance (13); in effector and memory T cells, sustained PD-1 signaling suppresses cytokine secretion and cytotoxic activity and contributes to the establishment of an exhausted phenotype during chronic antigen exposure (18). Moreover, PD-L1 also contributes to tolerance by promoting the differentiation of naïve CD4^+^ T cells into Foxp3^+^ induced regulatory T cells (iTreg), highlighting its essential role in peripheral tolerance (19).

Dysregulation of checkpoint signaling, particularly within the PD-1/PD-L1/PD-L2 axis, provides a mechanistic basis for the development of autoimmunity. Under physiological conditions, PD-1 signaling attenuates TCR- and CD28-driven pathways, limiting the expansion of autoreactive clones and maintaining peripheral tolerance. In systemic autoimmunity, when this inhibitory input is impaired—through genetic deficiency, altered ligand expression, or interference by soluble isoforms—CD4^+^ and CD8^+^ T cells exhibit sustained activation. This results in increased production of proinflammatory cytokines such as TNF-α, IFN-α, and IFN-γ, which promote the differentiation of autoreactive B cells and the persistence of long-lived memory clones. T follicular helper (Tfh) and T peripheral helper (Tph) cells, which frequently express high levels of PD-1, provide signals that promote B-cell activation and differentiation, thereby enhancing germinal center responses and driving the production of autoantibodies (20).

In AAV specifically, monocytes from patients exhibit reduced PD-L1 expression, indicating impaired inhibitory signaling from APC to T cells (21). In parallel, expansion of PD-1^hi^ CXCR5^−^ Tph subsets has been reported, providing augmented signals for B-cell activation and differentiation and thereby supporting autoreactive responses (22). In renal tissue, both PD-1 and PD-L1 expression are decreased, indicating a local loss of checkpoint control (23). Moreover, recent single-cell transcriptomic analyses have identified neutrophil subsets highly responsive to IFN-γ and TNF-α, cytokines that are typically elevated when checkpoint restraint is diminished. These neutrophils exhibit increased MPO and FcγR expression and are particularly susceptible to ANCA-induced activation and NET formation, linking checkpoint dysfunction to T-cell activation and neutrophil-driven vascular injury (24). In addition, Slot et al. (25) described genetic polymorphisms on PD-1 and CTLA4 than may also lead to T-cell hyperreactivity and contribute to the pathogenesis of AAV.

Similar alterations are reported in other autoimmune diseases. In systemic lupus erythematosus (26) and rheumatoid arthritis (27), circulating T cells often show increased PD-1 expression, which paradoxically associates with sustained activation and autoantibody production. In contrast, patients with type 1 diabetes or Sjögren’s syndrome, the frequency of PD-1^+^ T lymphocytes were found to be significantly decreased (27, 28). Despite these apparent differences, both scenarios reflect a common defect: inhibitory signaling through PD-1/PD-L1 is insufficient to maintain tolerance. Checkpoint knockout models clearly demonstrate their essential role in tolerance. PD-1–deficient mice develop chronic autoimmune disease, including lupus-like glomerulonephritis and arthritis (29), demonstrating that loss of this pathway lowers the threshold for lymphocyte activation and drives organ-specific autoimmunity (30).

The development of ANCA-associated vasculitis following immune checkpoint inhibitor therapy provides compelling evidence for the involvement of the PD-1 axis in disease pathogenesis (31). Understanding the precise role of the PD-1 axis in AAV could open avenues for targeted immunomodulatory strategies, potentially allowing the identification of patients at risk for relapse or those who may benefit from therapies such as rituximab.

### 2.2 CD28 axis

CD28 is a central co-stimulatory receptor of the CD28 family and an essential regulator of naïve T-cell activation. It is constitutively expressed on most CD4^+^ T cells and on a subset of CD8^+^ T cells. Through binding to the B7 ligands CD80 (B7-1) and CD86 (B7-2) on antigen-presenting cells, CD28 provides the co-stimulatory signals required for effective T-cell proliferation, survival, and cytokine production. This interaction is tightly controlled by the inhibitory receptor CTLA-4, which shares structural homology with CD28 and displays higher affinity for CD80 and CD86. By competing for these ligands, CTLA-4 restricts CD28-mediated activation and contributes to the regulation of T-cell responses (32). The balance between these activating and inhibitory inputs determines whether antigen-engaged T cells proceed toward effector differentiation or remain functionally restrained.

In ANCA-associated vasculitis, components of the CD28 pathway show disease-related modulation. The proportion of circulating CD4^+^CD28^+^ T cells is significantly higher in patients with low BVAS compared with those with high BVAS, suggesting that loss or down-modulation of CD28 expression may be associated with more active inflammatory disease (33). Consistent with functional relevance in AAV, T cells from patients with active granulomatosis with polyangiitis display enhanced proliferative responses following CD2/CD28 co-stimulation compared with healthy controls (34), supporting a role for heightened CD28-dependent signaling in pathogenic T-cell activation.

Evidence from other forms of vasculitis further reinforces the relevance of CD28-mediated co-stimulation in vascular inflammation. In a human artery–severe combined immunodeficiency mouse chimera model of giant cell arteritis, blockade of CD28 signaling disrupted T-cell metabolic fitness and markedly attenuated vessel wall inflammation and remodeling (35). Similarly, in Takayasu’s arteritis, active disease is associated with increased CD28 mRNA expression compared with inactive stages (36).

Finally, alterations of the CD28 axis have also been described in other systemic autoimmune diseases, providing additional context for its pathogenic relevance. In systemic lupus erythematosus, active disease is characterized by reduced proportions of CD4^+^CD28^+^ T cells together with elevated circulating levels of soluble CD28, which show a positive correlation with clinical disease activity and are associated with organ involvement, including lupus nephritis (37). These observations parallel the CD28 dysregulation observed in AAV and other vasculitis, supporting the concept that imbalance within the CD28/CTLA-4 pathway represents a shared mechanism of pathological T-cell activation across systemic autoimmune disorders.

### 2.3 CTLA-4 axis

CTLA-4 is an inhibitory receptor of the CD28 family that regulates early T-cell activation by competing with CD28 for the shared ligands CD80 and CD86 on antigen-presenting cells. Because CTLA-4 binds these ligands with higher affinity, it dampens co-stimulatory signaling, promotes peripheral tolerance, and supports the suppressive function of regulatory T cells. CTLA-4 also shapes helper T-cell differentiation by favoring Th1-driven responses while limiting Th2-associated cytokines—an effect that counters CD28-mediated activation (38). Given the predominance of Th1-type immunity in several autoimmune diseases, the balance between CD28 and CTLA-4 signaling is considered highly relevant to disease pathogenesis.

Alterations in both membrane-bound and soluble forms of CTLA-4 have been reported in AAV. At the cellular level, CTLA-4 expression is increased on circulating CD4^+^ T cells during active disease, and higher expression levels correlate with more severe clinical manifestations. However, despite this elevated baseline expression, T cells from GPA patients exhibit a reduced ability to further upregulate CTLA-4 following polyclonal stimulation, indicating an impaired capacity to mount appropriate inhibitory responses (39). Soluble components of this pathway are also disrupted: serum sCTLA-4 concentrations are lower in AAV patients than in healthy controls and decline further during remission, paralleling decreases in BVAS (33). These findings suggest that dysregulated CTLA-4 signaling—characterized by inadequate inducible inhibition and altered soluble mediator levels—may contribute to excessive T-cell activation and the loss of immune homeostasis in AAV.

### 2.4 ICOS axis

The inducible T-cell co-stimulator (ICOS, CD278) is a member of the CD28/CTLA-4 co-stimulatory receptor family that regulates T-cell activation and T–B cell collaboration. ICOS is inducibly expressed on activated CD4^+^ and CD8^+^ T cells, particularly on Tfh and Treg, where it promotes cytokine production, T-cell proliferation, and B-cell response. Its ligand, ICOSL (B7-H2, CD275), belongs to the B7 superfamily and is expressed on antigen-presenting cells, including B cells, monocytes, dendritic cells, and some non-hematopoietic cells such as endothelial and epithelial cells (40). The ICOS–ICOSL interaction activates downstream PI3K signaling, supporting germinal center formation, antibody production, and maintenance of long-lived plasma and memory cells (41). Given its central role in T–B collaboration, alterations in the ICOS pathway have been investigated as indicators of humoral immune activation in AAV.

Dysfunctional ICOS–ICOSL signaling is a well-recognized feature across multiple autoimmune diseases, providing mechanistic context for its involvement in AAV. In AAV, alterations in the ICOS–ICOSL co-stimulatory pathway have been documented at both cellular and soluble levels. Patients with MPO-AAV display an increased frequency of ICOS^+^ Tfh cells and a higher ICOS^+^/PD-1^+^ Tfh ratio compared with healthy controls (42), indicating an expansion of activated Tfh subsets capable of driving ANCA production through sustained B-cell stimulation. Consistent with these cellular abnormalities, serum concentrations of soluble ICOS (sICOS) are significantly elevated in AAV relative to controls (33), suggesting that systemic enhancement of ICOS signaling may contribute to dysregulated humoral immunity.

In RA, overexpression of ICOS and ICOSL within the synovium and in circulating immune cells promotes T-cell activation and Tfh-mediated B-cell responses. Increased frequencies of CD19^+^ICOSL^+^ B cells correlate with clinical disease activity and histopathologic damage (43), while therapeutic blockade of this pathway ameliorates inflammation and joint destruction in murine arthritis models (44). Similarly, in systemic lupus erythematosus (SLE), ICOS expression is significantly increased on both CD4^+^CD45RO^+^ and CD8^+^CD45RO^+^ T cells, with even higher levels observed in patients with lupus nephritis (45). Functional studies using a high-affinity anti-ICOS monoclonal antibody (JTA009) demonstrate that ICOS engagement enhances T-cell proliferation and cytokine secretion—including IFN-γ, IL-4, and IL-10—and promotes IgG anti–double-stranded DNA antibody production by autologous B cells (46).

Together, these findings highlight how exaggerated ICOS signaling supports aberrant T–B cell cooperation and amplifies humoral autoimmunity in AAV and likely contributes to the expansion of pathogenic Tfh cells, persistent B-cell activation, and the generation of ANCAs, underscoring ICOS–ICOSL signaling as a relevant pathway in disease pathogenesis.

### 2.5 CD40L axis

The CD40–CD40L interaction constitutes a major co-stimulatory pathway that regulates communication between T cells, B cells, and antigen-presenting or endothelial cells. CD40 is a member of the tumor necrosis factor receptor (TNFR) superfamily expressed on B cells, dendritic cells, macrophages, and various non-hematopoietic cells, whereas its ligand CD40L (CD154) is transiently expressed on activated CD4^+^ T cells and platelets. Engagement of CD40 by CD40L triggers downstream NF-κB signaling, induces cytokine release, promotes B-cell differentiation, immunoglobulin class switching, and upregulation of adhesion molecules, ultimately sustaining inflammatory and autoimmune responses (47).

In AAV, both membrane-bound and soluble CD40L (sCD40L) are elevated compared with healthy controls (48), highlighting a state of heightened costimulatory signaling. In contrast, serum concentrations of soluble CD40 (sCD40) did not differ significantly from controls but displayed strong correlations with indices of disease severity and renal dysfunction (33). As the CD40–CD40L axis is essential for T–B cell collaboration, germinal center formation, and the generation of high-affinity, in the context of AAV increased CD40L availability may potentiate aberrant B-cell activation and promote the production of ANCAs, a central driver of disease. Enhanced CD40 signaling can also amplify T-cell activation and inflammatory cytokine release, strengthening the proinflammatory milieu that contributes to endothelial injury and necrotizing vasculitis.

Findings from other autoimmune diseases provide strong mechanistic support for the pathogenic role of this pathway. In SLE, CD40L is overexpressed on immune cells (49), serum sCD40L levels correlate with disease activity and renal involvement (50), and transgenic overexpression of CD40L induces a lupus-like phenotype in mice (51), whereas blockade of the CD40–CD40L interaction reduces autoantibody production and glomerular inflammation (49). Similar patterns are observed in MS, where CD40 activation within B cells and the CNS promotes inflammation and demyelination, and inhibition of this pathway ameliorates disease in EAE models (52). These cross-disease observations reinforce the concept that sustained CD40–CD40L signaling drives chronic autoimmunity.

### 2.6 OX40 axis

The OX40–OX40L interaction represents a co-stimulatory signaling pathway that regulates T-cell activation, proliferation, and survival. OX40 (CD134), a member of the TNF receptor superfamily, is transiently expressed on activated CD4^+^ and CD8^+^ T cells, while its ligand OX40L (CD252, TNFSF4) is expressed on dendritic cells, B cells, activated T cells, mast cells, Langerhans cells, and vascular endothelial cells (12). Engagement of OX40 by trimeric OX40L enhances Th-cell polarization, sustains the function of regulatory and memory T cells, and promotes adhesion of activated T cells to the endothelium, thereby contributing to chronic inflammatory responses (53).

In AAV, increased expression of OX40 and an expansion of OX40^+^ T cells have been identified in both peripheral blood and inflamed tissues, where these cells predominantly produce TNF-α (54, 55). Although quantitative data on soluble OX40L in AAV remain limited, the available evidence suggests that activation of the OX40–OX40L pathway parallels disease activity and may contribute to the persistence of chronic T-cell responses that support ongoing vascular inflammation.

Beyond AAV, the involvement of the OX40–OX40L axis in other systemic autoimmune disorders further highlights its pathogenic potential. In RA, OX40 and OX40L are overexpressed in inflamed synovial tissue and circulating immune cells, where they enhance effector T-cell activation, promote Tfh differentiation, and sustain autoantibody production (56–58). Consistent with these cellular findings, soluble OX40L (sOX40L) is detectable in early RA and correlates with autoantibody positivity, supporting its value as a serological marker of humoral immune activation (57). Experimental blockade of the OX40–OX40L interaction in murine arthritis models markedly reduces inflammation and joint destruction, confirming its central role in disease pathogenesis (56). Similar mechanisms appear operative in SLE, where OX40L expression is increased on B cells and antigen-presenting cells, promoting Tfh differentiation and augmenting autoantibody production. Inhibition of OX40L in murine lupus models reduces disease severity and nephritis, reinforcing the contribution of this pathway to aberrant T–B cell collaboration and chronic autoimmunity (59, 60).

Taken together, these observations support the concept that heightened OX40–OX40L signaling contributes to the expansion of pathogenic T-cell populations, sustained B-cell help, and chronic inflammation in AAV, mirroring its established pathogenic roles in other autoimmune diseases.

### 2.7 LAG-3 axis

Lymphocyte activation gene-3 (LAG-3, CD223) is an inhibitory receptor structurally related to CD4 that binds MHC class II with higher affinity and modulates T-cell activation, proliferation, and effector function. LAG-3 contributes to the control of CD4^+^ and CD8^+^ T-cell responses, supports regulatory T-cell activity, and helps maintain immune homeostasis during persistent antigen exposure (61).

In AAV, LAG-3 remains a relatively underexplored immune checkpoint, and current evidence is largely restricted to its soluble form. Serum concentrations of soluble LAG-3 (sLAG-3) are elevated in patients compared with healthy controls (33), mirroring the increases reported for other inhibitory checkpoint molecules during active disease. These findings suggest that alterations in LAG-3 signaling may participate in the broader dysregulation of T-cell inhibitory pathways characteristic of AAV, although further studies are needed to clarify its mechanistic role.

### 2.8 TIM-3 axis

T-cell immunoglobulin and mucin-domain containing protein-3 (TIM-3) is a key inhibitory receptor expressed on a wide range of immune cells, including Th1 lymphocytes, cytotoxic T cells, Treg, and innate immune populations (61). Through interactions with ligands such as galectin-9, TIM-3 plays a critical role in limiting Th1-mediated inflammation and modulating myeloid cell activation.

In AAV, both cellular and soluble components of the TIM-3 pathway are altered in a disease-associated manner. At the cellular level, MPO-AAV patients display markedly reduced TIM-3 expression on dendritic cells. Notably, experimental blockade of TIM-3 further enhances NET-mediated dendritic cell cytokine production, indicating that loss of TIM-3 function facilitates NET-driven inflammation (62). These observations suggest that impaired TIM-3 signaling contributes to the amplification of innate and adaptive immune responses in AAV, promoting vascular inflammation and tissue damage.

### 2.9 BTLA axis

B- and T-lymphocyte attenuator (BTLA) is an inhibitory receptor expressed on T cells, B cells, and dendritic cells, which interacts with its ligand, herpesvirus entry mediator (HVEM), to deliver negative regulatory signals that restrain immune activation. BTLA signaling is essential for maintaining peripheral tolerance, limiting T-cell proliferation, and dampening proinflammatory cytokine production (63).

Evidence on BTLA as a co-inhibitory pathway in human autoimmune diseases remains limited, and its pathogenic relevance is yet to be fully established. However, impaired BTLA–HVEM signaling has been hypothesized to diminish inhibitory checkpoint control, thereby facilitating sustained T- and B-cell activation. Available data in SLE support this concept, as reduced BTLA levels have been reported in patients and have been associated with disease progression (64). In AAV specifically, Werner et al. (65) reported reduced BTLA expression in circulating CD3^+^CD4^−^CD8^−^ double-negative T cells from AAV patients in remission compared with healthy controls, and this reduction was associated with disease activity and relapse rate. Moreover, BTLA stimulation using agonistic antibodies suppresses T-cell proliferation and decreases Th17 activity in both patients and controls, supporting a potential therapeutic role for BTLA pathway modulation in AAV. Notably, recent renal transcriptomic profiling studies have reported upregulation of BTLA in affected kidney tissue compared with healthy controls (66). In addition, soluble BTLA (sBTLA) levels were reported to significantly decrease with declining BVAS in AAV patients, supporting its potential utility as a dynamic marker of disease activity (33).

These findings suggest that BTLA dysregulation may contribute to disease pathogenesis in AAV. Further studies are needed to characterize both cellular and soluble components of the BTLA axis and to explore its potential as a therapeutic target.

### 2.10 CD27 axis

CD27 is a co-stimulatory receptor of the tumor necrosis factor (TNF) receptor superfamily, expressed primarily on T cells, memory B cells, and subsets of natural killer cells. Its interaction with CD70 provides critical signals for T-cell activation, B-cell differentiation, and the generation of long-lived plasma cells. CD27–CD70 signaling is essential for effective adaptive immune responses, but sustained or dysregulated activation can contribute to chronic inflammation and autoimmunity (67).

In AAV, alterations in the CD27 axis have been less extensively studied, but available evidence suggests a potential role in disease pathogenesis. Increased frequencies of CD27^+^ T cells and CD27^+^ memory B cells could facilitate enhanced B-cell help and support ANCA production, thereby supporting persistent humoral autoimmunity, as circulating CD27^hi^ CD38^hi^ B cells have been identified as direct precursors of autoantibody-producing plasma cells. Focusing on B cells, Wang et al. described a significant increase in CD27^hi^ CD38^hi^ B cells during the acute phase of the disease, which additionally correlated with renal involvement (68). Although data remain limited, recent renal transcriptomic profiling studies further support the relevance of this pathway at the target organ level, reporting upregulation of CD27 in affected kidney tissue compared with healthy (66). In parallel, serum concentrations of soluble CD27 (sCD27) are elevated in patients compared with healthy controls (33). Investigation of both cellular and soluble components of this pathway may provide insights into disease activity and identify novel therapeutic opportunities.

## 3 Immune checkpoint pathway components in AAV: utility as disease biomarkers

Despite advances in clinical management, currently available biomarkers for AAV remain limited. ANCA titers, the Birmingham Vasculitis Activity Score, and renal function indices provide clinically useful information but do not reliably predict relapse, long-term outcomes, or therapeutic response. This gap underscores the need for biomarkers that more accurately reflect the underlying immune dysregulation.

In this setting, immune checkpoint molecules—particularly their soluble isoforms—have emerged as promising candidates. Alterations in their expression, quantify the imbalance between effector activation and inhibitory control, providing measurable correlates of immune dysregulation detectable in blood and affected tissues. Longitudinal cohort studies have identified reproducible associations between soluble checkpoint profiles and disease activity, renal involvement, and relapse risk. Next, we describe the most promising biomarkers related to checkpoint pathway activity and relapse in AAV identified to date.

### 3.1 PD-1/PD-L1/PD-L2 axis

Multiple studies have reported dysregulation of the PD-1 axis in AAV. As described before, at the cellular level, active AAV is characterized by increased PD-1 expression on circulating Tph cells involved in extrafollicular B-cell activation, with PD-1^hi^ Tph frequencies decreasing after immunosuppressive therapy (22). In parallel, a reduced availability of PD-1 ligands is observed in myeloid cells: circulating monocytes and neutrophils show diminished PD-L1 expression, which correlates with higher ANCA titers and greater disease severity (21). Importantly, reduced PD-L1 expression—particularly on neutrophils—can persist even during clinical remission, indicating incomplete restoration of immune checkpoint regulation (23). Consistent with these systemic findings, renal biopsies from patients with pauci-immune glomerulonephritis reveal a profound disruption of the PD-1/PD-L1 inhibitory axis. Both tubular and glomerular compartments show marked loss of PD-L1 expression (23), generating a renal microenvironment with limited inhibitory signaling that may favor the infiltration and activation of CD4^+^ T cells, B cells, neutrophils, and macrophages. In parallel, tubulointerstitial PD-1 expression is reduced in renal AAV and correlates with the presence of active glomerular and interstitial lesions (69), supporting a role for local checkpoint dysregulation in sustaining renal inflammation.

Soluble elements of this pathway also exhibit disease-associated variation, as serum sPD-1 concentrations are elevated in active AAV and correlate with BVAS and systemic inflammatory markers (33). In addition, it has been described that sPD-L1 increases during active disease and decreases with treatment, whereas sPD-L2 shows the opposite pattern, lower during activity and higher in remission (33). Extending these observations to the urinary compartment, urinary PD-1 and PD-L2 concentrations are reduced in patients with AAV compared with healthy controls, reaching their lowest levels during acute disease and partially recovering during remission, suggesting that local renal checkpoint dynamics are also reflected in urine biomarkers (70).

Taken together, these findings highlight a broad breakdown of PD-1–mediated inhibitory signaling in AAV, with increased PD-1 on pathogenic T-cell subsets and reduced PD-L1 across immune and renal compartments, a pattern that may serve as a useful indicator of immunologic activity and disease flare. Comparable alterations within the PD-1 axis have also been described in SLE, underscoring that the impairment of this axe represents a recurrent immunological disturbance across systemic autoimmune diseases (71–73).

### 3.2 Other checkpoint molecules

Increased levels of several immune checkpoint molecules are associated with the presence and severity of disease activity. Serum concentrations of soluble CD28 (sCD28) are elevated compared with healthy controls and correlate with BVAS and systemic inflammatory indices (33). In contrast, serum concentrations of soluble CD40 (sCD40) are not significantly different from controls but show strong correlations with indices of disease severity and renal dysfunction (33). sTIM-3 levels correlate strongly with BVAS and renal parameters and decrease with treatment, confirming its value as a reliable and dynamic marker (33). Taken together, these findings indicate that circulating checkpoint molecules—particularly sCD28 and sTIM-3—may serve as useful biomarkers for assessing disease activity and organ involvement in AAV.

The evaluation of checkpoint molecule levels may be useful in assessing the response to rituximab. In the study conducted by Gamreith et al. (74) among patients receiving rituximab induction therapy the combination of lower sLAG-3 and higher sCD27 predicted therapy failure. In another study, Miyazaki et al. (75) reported that patients with a higher proportion of CD27^−^ B cells are at greater risk of poor remission, but treatment with rituximab can significantly improve their clinical outcomes compared to conventional therapy. This highlights CD27^−^ B cells as a potential biomarker for identifying patients who may benefit most from rituximab.

Some authors have also explored the role of checkpoint molecules as markers of relapse risk. In patients treated with rituximab, a multimarker strategy combining sTIM-3, sCD27, and sBTLA4 showed that lower levels of these molecules predicted relapse (74). In the same line, frequently relapsing EGPA has been associated with increased percentages of CD80^+^ B cells during active disease (76), indicating enhanced ligand availability in flare conditions.

## 4 Immune checkpoint pathways as treatment targets in AAV

Only one targeted therapy acting on immune checkpoint pathways—abatacept, a CTLA-4 fusion protein—has been clinically evaluated in AAV. The therapeutic relevance of other checkpoint molecules in AAV therefore remains largely speculative, based primarily on mechanistic considerations and clinical experience derived from other autoimmune diseases. Nevertheless, accumulating evidence from related conditions suggests that additional immune checkpoint–targeted strategies may ultimately prove valuable in AAV.

Abatacept consists of the ligand-binding domain of CTLA-4 fused to a modified IgG1 Fc domain. By engaging CD80 and CD86, abatacept prevents CD28-mediated co-stimulation and thereby inhibits T-cell activation. Based on this rationale, Langford et al. (77) conducted an open-label study in patients with non-severe relapsing GPA, demonstrating that abatacept was well tolerated and associated with a high rate of disease remission.

However, a subsequent randomized trial yielded contrasting results. In patients with relapsing, non-severe GPA, the addition of abatacept to glucocorticoids did not reduce the risk of relapse, severe disease worsening, or failure to achieve remission. Furthermore, abatacept showed no statistically significant benefit over placebo in key secondary outcomes, including relapse severity, time to full remission, duration of glucocorticoid-free remission, prevention of organ damage, or patient-reported quality of life (78).

Beyond CTLA-4, PD-1 agonism has emerged as a highly promising therapeutic approach in autoimmune diseases, supported by a strong mechanistic rationale and encouraging early clinical data. In rheumatoid arthritis, the PD-1 agonist antibodies peresolimab and rosnilimab have demonstrated clinically meaningful reductions in disease activity in patients refractory to conventional therapy (12). Rosnilimab is also under investigation in alopecia areata, an autoimmune disorder driven by autoreactive cytotoxic CD8 + T cells that target hair follicles (79). More recently, the PD-1 agonist antibody JNJ-67484703 was well tolerated and showed evidence of biologic and clinical activity in a proof-of-mechanism phase Ib study in adults with active rheumatoid arthritis (80), supporting further evaluation of PD-1 agonism in larger trials.

Interruption of the OX40 axis has been investigated in other autoimmune conditions, including psoriasis, ulcerative colitis, and atopic dermatitis (81–83). However, OX40 blockade also inhibits Tregs, which may be counterproductive in AAV, as Treg impairment could potentially trigger disease flares (12). It remains speculative whether combining OX40 blockade with other immunosuppressants could mitigate this effect and provide additional benefit (84).

Targeting the ICOS–ICOSL pathway has been investigated in SLE. AMG 557, a human IgG2 monoclonal antibody that selectively inhibits ICOS–ICOSL interactions, has been reported to improve inflammatory responses in patients with SLE (85, 86). Given the role of this pathway in T-helper cell activation and differentiation, its modulation may also be relevant in autoantibody-mediated diseases such as AAV, suggesting potential translatability of this approach.

Finally, *in vitro* evidence supports the potential utility of inhibiting CD40 signaling in AAV. CD40 promotes autoreactive B-cell activation and ANCA production through NF-κB–dependent pathways. Its inhibition could therefore suppress pathogenic B-cell responses and reduce autoantibody production (87). However, clinical studies targeting this pathway in rheumatoid arthritis and SLE have yielded mixed results, leaving its therapeutic benefit in AAV uncertain (88, 89).

In summary, abatacept remains the only immune checkpoint–directed therapy formally tested in AAV, and its lack of efficacy in randomized trials should not discourage further investigation into the role of T cells and co-stimulatory pathways in disease pathogenesis. Rather, these findings underscore the complexity of immune regulation in AAV and the need for refined therapeutic strategies. As our understanding of T-cell co-stimulatory and co-inhibitory networks expands, well-designed clinical trials will be essential to determine whether targeting these pathways can yield meaningful therapeutic advances.

## 5 Discussion and future perspectives

Immune checkpoint pathways play a central role in maintaining peripheral tolerance, and accumulating evidence supports their involvement in the immunopathogenesis of ANCA-associated vasculitis. Beyond the well-established contribution of ANCAs and innate immune activation, AAV is characterized by sustained T-cell activation and dysregulated T–B cell collaboration, in which an imbalance between co-stimulatory and co-inhibitory signaling may contribute to persistent inflammation, autoantibody production, and end-organ injury. In this context, both membrane-bound and soluble checkpoint components have emerged as measurable correlates of immune dysregulation, with potential value as biomarkers complementing clinical and serological assessment.

Beyond circulating biomarkers, recent renal transcriptomic analyses in AAV provide additional tissue-level evidence supporting the relevance of immune checkpoint pathways in target organ inflammation. In affected kidneys, several checkpoint-related genes were identified among the differentially expressed transcripts compared with healthy controls, including CTLA4, CD86, TIGIT, LAG3, CD27, PD-L2, and BTLA, together with enrichment of checkpoint-related immune pathways. Importantly, these immune signatures were linked to kidney outcomes, and a non-linear (“V-shaped”) association was described for multiple immunologic pathways including T-cell checkpoint activity where both low and high pathway activity were associated with improved renal survival, whereas intermediate activity correlated with worse outcomes (66). This observation suggests that checkpoint pathway activity in AAV may reflect distinct immunologic states with different prognostic implications, highlighting the need for longitudinal, tissue-informed studies to define how these regulatory programs relate to injury, repair, and treatment response.

To date, abatacept remains the only immune checkpoint–directed therapy evaluated in AAV in a randomized clinical trial setting. While early open-label data suggested potential benefit in relapsing, non-severe GPA, the subsequent randomized trial did not demonstrate improvement in primary or key secondary clinical outcomes. Several factors may explain these negative findings. First, nonsevere nature of the patient population, in whom disease activity is often subtle, fluctuating, and difficult to distinguish from damage or treatment withdrawal effects, potentially limiting the ability to detect a therapeutic signal. In such a context, even biologically active agents may confer only modest benefits that fall below clinically meaningful or statistically detectable thresholds. Second, the dosing and mechanism of action of abatacept may be insufficient to adequately suppress the complex and redundant immune pathways driving relapse in AAV, particularly when compared with therapies that directly deplete pathogenic B cells. Indeed, direct targeting of CTLA-4 may be intrinsically limited by rapid CTLA-4 endocytosis, raising the possibility that alternative strategies more directly inhibiting CD28–CD80/CD86 interactions could be more effective. Third, genetic variability may play a role, as polymorphisms in the *CTLA4* gene have been associated with GPA but not with MPA in Japanese populations, indicating potential disease- and race-specific differences in pathway relevance. Finally, the study was powered to detect only relatively large treatment effects; thus, smaller but potentially meaningful benefits may have gone undetected, raising the question of whether modest biologic effects would translate into clinically relevant outcomes. These findings help explain why checkpoint-targeted therapies have not yet entered routine clinical practice in AAV: despite strong mechanistic rationale implicating T cells in disease pathophysiology, clinical efficacy has not been consistently demonstrated in randomized trials, and identifying the appropriate patient subsets, disease stages, and combination strategies remains challenging.

Beyond CTLA-4–based modulation, PD-1 agonism has emerged as an attractive immunotherapeutic strategy in autoimmune diseases, supported by a strong mechanistic rationale and early efficacy signals in rheumatoid arthritis. Nevertheless, translation into AAV requires careful consideration of safety and disease biology. Checkpoint agonism may carry a risk of over-immunosuppression, potentially increasing susceptibility to infections, which already represent a major cause of morbidity and mortality in AAV under conventional immunosuppressive regimens. Conversely, paradoxical immune effects have been reported in other contexts of checkpoint manipulation, underscoring that altering these regulatory pathways may have non-linear and context-dependent consequences. Given the heterogeneous immune landscape of AAV—where innate effector mechanisms and adaptive responses interact dynamically across disease stages—checkpoint modulation may produce divergent outcomes depending on timing, prior treatments, tissue involvement, and individual immune profiles. Accordingly, future efforts should prioritize biomarker-guided stratification, robust safety monitoring, and carefully designed trials that incorporate mechanistic endpoints to define the conditions under which checkpoint-directed immunomodulation may be both effective and safe.

Collectively, current data position immune checkpoints as key regulators of AAV pathophysiology and as a promising frontier for improved disease monitoring and immune-targeted interventions. Nevertheless, evidence remains incomplete, and further studies are required to clarify how checkpoint dysregulation evolves across disease stages and organ involvement and to determine which pathways may be clinically actionable in AAV.
