# Supplementary material for: Immune checkpoint imbalance in ANCA-associated vasculitis: insights into disease activity and precision immunotherapy
Source: Front Med (Lausanne). 2026 Mar 2;13:1773241. doi: 10.3389/fmed.2026.1773241 (PMC12990133; doi:10.3389/fmed.2026.1773241)
Supplement: Supplementary file 1 [file Table_1.DOCX]

Supplementary Material

# Supplementary Box S1

The literature discussed in this review was identified through a targeted search of PubMed, covering studies published up to January 2026. Search terms included combinations of disease-related keywords (“ANCA-associated vasculitis”) and immune checkpoint–related terms (“immune checkpoints,” “PD-1,” “PD-L1,” “PD-L2,” “CTLA-4,” “CD28,” “ICOS,” “OX40,” “CD40,” “CD40L,” “TIM-3,” “LAG-3,” “BTLA,” “CD27”). Reference lists of relevant articles were manually screened to identify additional studies of interest. Human clinical cohorts and translational studies constitute the main focus of this review, with murine preclinical models included only when directly informative for immune checkpoint biology in AAV. Clinical trials were identified through a targeted search of the ClinicalTrials.gov database using immunotherapy-related keywords and disease-specific terms.
